# Mass Spectrometric Analysis of the Active Site Tryptic Peptide of Recombinant *O*^6^-Methylguanine-DNA Methyltransferase Following Incubation with Human Colorectal DNA Reveals the Presence of an *O*^6^-Alkylguanine Adductome

**DOI:** 10.1021/acs.chemrestox.3c00207

**Published:** 2023-11-20

**Authors:** Rasha Abdelhady, Pattama Senthong, Claire E. Eyers, Onrapak Reamtong, Elizabeth Cowley, Luca Cannizzaro, Joanna Stimpson, Kathleen Cain, Oliver J. Wilkinson, Nicholas H. Williams, Perdita E. Barran, Geoffrey P. Margison, David M. Williams, Andrew C. Povey

**Affiliations:** † Epidemiology and Public Health Group, Division of Population Health, Health Services Research and Primary Care, School of Health Sciences, Faculty of Biology, Medicine and Health, University of Manchester, Manchester M13 9PL, U.K.; ‡ Department of Chemistry and Manchester Institute of Biotechnology, University of Manchester, Manchester M1 7DN, U.K.; § Centre for Chemical Biology, Department of Chemistry, Sheffield Institute for Nucleic Acids, University of Sheffield, Sheffield S3 7HF, U.K.

## Abstract

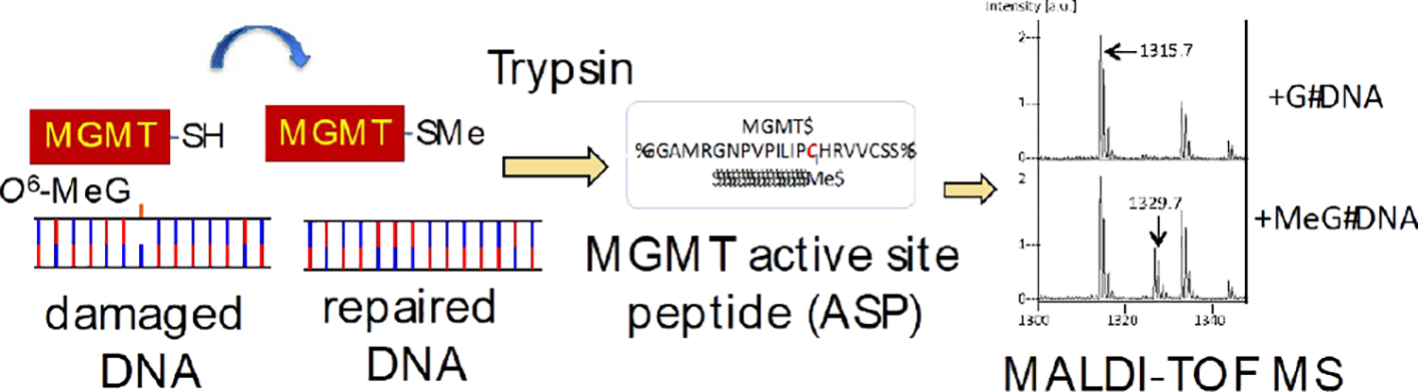

Human exposure to DNA alkylating agents is poorly characterized, partly because only a limited range of specific alkyl DNA adducts have been quantified. The human DNA repair protein, *O*^6^-methylguanine *O*^6^-methyltransferase (MGMT), irreversibly transfers the alkyl group from DNA *O*^6^-alkylguanines (*O*^6^-alkGs) to an acceptor cysteine, allowing the simultaneous detection of multiple *O*^6^-alkG modifications in DNA by mass spectrometric analysis of the MGMT active site peptide (ASP). Recombinant MGMT was incubated with oligodeoxyribonucleotides (ODNs) containing different *O*^6^-alkGs, Temozolomide-methylated calf thymus DNA (Me-CT-DNA), or human colorectal DNA of known *O*^6^-MethylG (*O*^6^-MeG) levels. It was digested with trypsin, and ASPs were detected and quantified by matrix-assisted laser desorption/ionization-time-of-flight mass spectrometry. ASPs containing *S*-methyl, *S*-ethyl, *S*-propyl, *S*-hydroxyethyl, *S*-carboxymethyl, *S*-benzyl, and *S*-pyridyloxobutyl cysteine groups were detected by incubating MGMT with ODNs containing the corresponding *O*^6^-alkGs. The LOQ of ASPs containing *S*-methylcysteine detected after MGMT incubation with Me-CT-DNA was <0.05 pmol *O*^6^-MeG per mg CT-DNA. Incubation of MGMT with human colorectal DNA produced ASPs containing *S*-methylcysteine at levels that correlated with those of *O*^6^-MeG determined previously by HPLC-radioimmunoassay (*r*^2^ = 0.74; p = 0.014). *O*^6^-CMG, a putative *O*^6^-hydroxyethylG adduct, and other potential unidentified MGMT substrates were also detected in human DNA samples. This novel approach to the identification and quantitation of *O*^6^-alkGs in human DNA has revealed the existence of a human DNA alkyl adductome that remains to be fully characterized. The methodology establishes a platform for characterizing the human DNA *O*^6^-alkG adductome and, given the mutagenic potential of *O*^6^-alkGs, can provide mechanistic information about cancer pathogenesis.

## Introduction

Alkylating agents (AAs) are known human mutagens and carcinogens whose effects are largely mediated by the formation of alkyl adducts in DNA.^1−3^ The mutational landscape observed in patients with malignant melanomas and glioblastoma multiformes following treatment with the chemotherapeutic methylating agent, Temozolomide, consists primarily of G-A transitions attributed to the generation of *O*^6^-methylguanine (*O*^6^-MeG) in DNA.^4^ A similar mutational signature has recently been described in colorectal cancer implicating AA exposure as a causal factor in this disease.^5^*O*^6^-MeG and other *O*^6^-alkylguanine (*O*^6^-alkG) adducts are repaired by the DNA repair protein, *O*^6^-methylguanine-DNA methyltransferase (MGMT), which provides protection against the toxic, mutagenic, and carcinogenic effects of AA exposure.^6,7^ Increased methylation of CpG islands in the MGMT promoter region has been described in human tumors^8,9^ and is associated with G-A transitions in colorectal,^10^ lung,^11^ and brain tumors^12^ and the Temozolomide-induced mutational signature.^13^ Furthermore, MGMT promoter methylation improves overall survival in glioblastoma patients treated with Temozolomide.^14^ As promoter methylation downregulates MGMT expression,^12,15^ these results are entirely consistent with persistence of *O*^6^-alkGs in DNA as a cause of toxicity and mutational events and provide compelling evidence that AAs are involved in the etiology of some cancers.

In addition to this indirect evidence for the presence of *O*^6^-alkGs in human DNA, there is increasing direct evidence for their presence in human DNA as described in several reviews^16−18^ of alkyl DNA adductomes. Thus, *O*^6^-MeG,^19^*O*^6^-ethyl (*O*^6^-EtG),^20^*O*^6^-propyl (*O*^6^-PrG),^21^*O*^6^-butyl (*O*^6^-BuG),^21^ and *O*^6^-carboxymethyl (*O*^6^-CMG)^22,23^ as well as 7-alkylguanines^16^ and methyl DNA phosphate adducts^24^ have all been detected in human DNA. The presence of a wider spectrum of alkyl adducts is not surprising given the wide range of AAs present in the human environment^25^ and that AAs can also be generated endogenously^26^ from the many varied and abundant dietary and luminal amines and other substrates.^27,28^ These processes likely result in AA exposure that significantly increases mutational and cancer risk by the formation of a range of associated *O*^6^-alkGs.^29^ Previous studies have largely focused on quantifying the presence of *O*^6^-MeG in human DNA using radioimmunoassays (RIA),^19^^32^P-postlabeling,^20,30^ and mass spectrometry (MS), e.g., high-resolution gas chromatography-MS with selected ion recording^21^ and ultrahigh-performance liquid chromatography-high resolution MS/MS,^23^ approaches with differing sensitivities and specificities. Some caution may be needed in some cases, for example, antibodies may recognize a range of *O*^6^-alkGs and hence the levels of *O*^6^-MeG may be overestimated if the *O*^6^-MeG is not completely separated from other possible *O*^6^-alkGs before the immunoassay quantitation. MS, in particular, has been routinely used in clinical settings and is increasingly used to detect with high sensitivity and specificity a wide range of different DNA adducts using relatively simple procedures that do not need radioactive materials or antibodies.^31−33^

In the present paper, we describe a novel method to assess the *O*^6^-alkG adductome by the MS analysis of alkylated MGMT active site peptides (ASPs) following *in vitro* incubation of MGMT with extracted DNA, which results in the irreversible transfer of the alkyl group from the *O*^6^ position of the modified guanine bases to the active site cysteine residue in MGMT.

## Materials and Methods

### Samples

A cross-sectional study of patients presenting with colorectal carcinoma at hospitals within Greater Manchester, U.K., was undertaken. Patients were included if they were undergoing surgery for treatment, and human colorectal tumor (*n* = 10; obtained from colorectal carcinoma tissue) and macroscopically normal (*n* = 3; taken ∼5 cm from the tumor edge) tissues were obtained from individuals (six men, two women, and two participants of unknown sex). The age of the eight individuals was 69 ± 14 (mean ± SD). DNA was extracted by a phenol/chloroform procedure and analyzed for *O*^6^-MeG by an HPLC-radioimmunoassay (RIA) using a [^3^H]-*O*^6^-methyldeoxyguanosine tracer and mouse monoclonal α-*O*^6^-MedG following Aminex chromatography,^19^ and the remaining DNA was stored at −80 °C until it was analyzed in the present study. Ethical approval was obtained from East Midlands-Derby Research Ethics Committee, Health Research Authority, NHS (REC reference: 15/EM/0505).

### Materials

Synthetic methylated (GNPVPILIPMe-CHR) MGMT-ASP and light and heavy isotope (^13^C^15^N proline)-labeled methylated MGMT-ASP corresponding to positions 136–147 of MGMT were purchased from Cambridge Research Biochemicals, Cleveland, U.K. Other chemicals used in this work were purchased from Sigma-Aldrich (Poole, Dorset, U.K.) unless otherwise stated. Calf thymus (CT) DNAs containing various levels of *O*^6^-MeG (0.050, 0.125, 0.250, and 0.50 pmol/mg-CT-DNA) were prepared by incubating Temozolomide with CT-DNA, and levels were determined by a competitive radioisotope-based assay involving preincubation of incrementally increasing amounts of the Temozolomide-methylated CT DNA with a fixed amount of MGMT and then post incubation with excess *N*-[^3^H]methyl-*N*-nitrosourea (Hartmann Radiochemicals: specific radioactivity 80 Ci/mmole) methylated CT DNA. The decrease in the amount of radioactivity transferred to the MGMT was used to determine the amount of *O*^6^-MeG in the Temozolomide-methylated DNA, as described previously.^34^

### Expression and Purification of MGMT

Human MGMT was expressed as a maltose-binding protein (MBP) fusion protein from pMAL-2c expression vector constructs and affinity-purified using amylose resin (New England Biolabs Inc., USA) essentially as described previously.^35^ For some studies, the MBP-MGMT fusion protein was cleaved with factor Xa, and MGMT was purified using DEAE-sepharose ion exchange chromatography. Human MGMT was also expressed as a hexahistidine (His) fusion protein from pQE30Xa (Qiagen) and purified by nickel affinity chromatography using a complete His-Tag purification resin (Sigma-Aldrich). MGMT activity was subsequently assayed by measuring the transfer of [^3^H] from *N*-[^3^H]-methyl-*N*-nitrosourea (Hartmann, Germany; specific activity 80 Ci/mmol) methylated CT-DNA to the MGMT fusion protein.^36^ In addition, for the Vion IMS QToF analysis, MGMT was synthesized by GeneArt (ThermoFisher), cloned into pNic28-Bsa4 linearized with BsaI-HF (NEB) using In-Fusion ligation independent cloning. Competent BL21(DE3) cells (NEB) were transformed with the vector, His-MGMT expressed, and purified by nickel affinity chromatography.

### Synthesis of Oligodeoxyribonucleotides Containing a Single *O*^6^-alkG Adduct

*O*^6^-alkG-containing 12- or 23 mer-oligodeoxyribonucleotides (ODNs) that contained a single *O*^6^-alkG adduct in the following sequences, 5′ -SIMA-GCC ATG XCT AGTA or 5′-GAA CTY CAG CTC CGT GCT GGC CC-3′, were synthesized as described.^37−40^ X was unmodified G, *O*^6^-MeG, *O*^6^-EtG, *O*^6^-PrG, *O*^6^-hydroxyethyl (*O*^6^-HOEtG), *O*^6^-benzylG (*O*^6^-BnG), *O*^6^-pyridyl-oxobutylG (*O*^6^-pobG), 2,6-diaminopurine, *O*^6^-aminoethylG, N6-hydroxypropyl-2,6-diaminopurine, or *O*^6^-methyladamantylG, and Y was unmodified G or *O*^6^-MeG or *O*^6^-CMG. Modified 12-mer ODNs were characterized by ESI-MS as previously described for the 12-mers^39^ and 23-mers in Figure s1.

Control ODNs (with unmodified G bases) as well as complementary ODNs were synthesized by Sigma-Aldrich, U.K. Single-stranded ODNs were annealed to equimolar amounts of the ODN complement by heating to 95 °C in 50 mM NaCl for 20 min and then cooling slowly to room temperature for >1 h. ODNs were stored at −20 °C.^40^

### Preparation of MGMT Tryptic Peptides following Incubation of MGMT with ODNs and Methylated CT-DNA

In a typical assay, double-stranded 23-mer ODNs (20 pmol) containing G, *O*^6^-MeG, or *O*^6^-CMG or Temozolomide-methylated CT DNA (Me-CT-DNA) were incubated with MBP-MGMT (2 pmol by activity) for 6 h at 37 °C in IBSA buffer (1 mg/mL BSA in 50 mM Tris-HCl pH 8.3 containing 1 mM EDTA and 1 mM Tris(2-carboxyethyl)phosphine hydrochloride (TCEP)). Trypsin (ratio of MBP-MGMT:trypsin, 20:1) was added, the sample was incubated overnight (18 h) at 37 °C with shaking, and then 1% formic acid was added to give a final concentration of 0.1%. MGMT tryptic peptides were desalted, concentrated using Millipore C18-Ziptips (Merck Millipore, Ireland) according to manufacturer’s instructions, and eluted in 5 μL of 0.1% formic acid in 50% acetonitrile/water.

### Preparation of MGMT Tryptic Peptides following Incubation of MGMT with Human DNA

His-MGMT (30 pmol by activity) was incubated with 2 mg of human colorectal DNA for 6 h at 37 °C in 50 mM Tris-HCl pH 8.3 containing 1 mM EDTA and 2 mM TCEP on a shaker incubator. Prewashed Ni-coated magnetic beads (PureProteome Magnetic Beads, Merck Millipore, U.K.) were resuspended in equilibration buffer, vortex-mixed, added to the His-MGMT/DNA solution, and incubated at 4 °C overnight on a rotor mixer (Blood Tube Rotator, SB1, Stuart Scientific, U.K.). The sample was then centrifuged (Fisher Scientific accuSpin Microcentrifuges), and the supernatant was aspirated. Beads were resuspended in 40 μL of buffer containing 50 mM sodium phosphate, 300 mM sodium chloride, and 300 mM imidazole, pH 8. Trypsin was added (as above), and the sample was incubated overnight (18 h) at 37 °C with shaking. Formic acid (1%) was added to give a final concentration of 0.1%, and the tryptic peptides were desalted and concentrated using Millipore C18-Ziptips and then spiked with 250 fmol of ^13^C^15^N proline-labeled methylated ASP internal standard.

### MALDI-ToF MS Analysis of MGMT Active Site Peptides and Data Acquisition

Tryptic peptides arising from MGMT, MBP-MGMT, and His-MGMT were spotted on a MALDI plate together with a saturated α-cyano-4-hydroxycinnamic acid (Fluka, Buchs, Switzerland) matrix solution (10 mg/mL in 50% ethanol/acetonitrile). The MALDI-ToF was calibrated by using a J67722 MALDI certified mass spec calibration standard (Alfa Aesar, U.K.). Spectra were acquired over the mass to charge ratio (*m/z)* range 800–2300 using a Bruker (Germany) Ultraflex II operating at 30% laser intensity and 1000 laser shots per spectrum in reflectron positive ion mode. A signal/noise >10 was required for identification of detected alkylated peptide ions. Peak areas (PAs) of chosen peptides (methylated MGMT-ASP and internal standard) were measured using FlexAnalysis software (Bruker, Germany). Label-free quantitation of *O*^6^-CMG adducts was carried out by comparing the PA of carboxymethylated ASP to that of methylated ASP generated from known amounts of the appropriate *O*^6^-alkG-containing ODNs and applying this ratio to the PAs of carboxymethylated ASPs found after incubating MGMT with colorectal DNA.

### Vion IMS QTof Analysis and Data Acquisition of His-MGMT Active Site Peptides

Control (G) and *O*^6^-MeG-containing single-stranded 23-mer ODNs (*O*^6^-MeG) (37.5 nmol) were incubated with 50 pmol of His-MGMT in 50 mM Tris-HCl pH 8.3 containing 1 mM EDTA and 5 mM DTT for 90 min at 37 °C. In-solution digestion was performed on the samples with 50 mM DTT, 14 mM iodoacetamide, and trypsin (ratio of His-MGMT:trypsin ≤ 12:1) was added and incubated overnight (18 h) at 37 °C. After in-solution trypsin digestion and concentration, one sample of the single-stranded control ODN (G) was spiked with 112 nmol of synthetic methylated ASP. The samples were dried using an SP Genevac miVac Sample Concentrator and then dissolved in water containing 0.05% acetonitrile and 0.1% formic acid.

Tryptic peptides were resolved by ultraperformance liquid chromatography using an ACQUITY UPLC I-Class System and a 100 × 2.1 mm Hypersil GOLD C18 3 μm column (ThermoFisher) in tandem with a quadruple time-of-flight mass spectrometer (Vion IMS QToF, Waters). Water containing 0.1% formic acid was used as mobile phase A, and acetonitrile containing 0.1% formic acid was used as mobile phase B. The flow rate was 0.2 mL/min, and the total elution time was 60 min. The elution gradient program was as follows: 0 to 3 min, 99% A; 3 to 53 min, 99 to 80% A; 53 to 55 min, 80 to 20% A; 55 to 57 min, 20% A; 57 to 58 min, 20 to 99% A; 58 to 60 min, 99% A. Electrospray ionization was carried out in positive ion mode with an ion source temperature of 120 °C. The mass scan range was from 105 to 2000 *m*/*z*, with a scan time of 0.250 s. LockSpray solution containing the peptide leucine/enkephalin was analyzed every 2 min to adjust mass calibration of the instrument during analysis. Data were collected in MS^E^ mode^41^ where the instrument alternated between low (6 eV for precursor ion collection) and high (15–40 eV ramp for fragment ion collection) collision energies throughout the entire chromatographic run. Data were analyzed using UNIFI software version 1.9.4.053 (Waters Corporation); the selected amino acid modifiers were methyl (cysteine), carbamidomethyl (cysteine), oxidation (methionine), and deamidation (asparagine).

## Results

### MS Analysis of MGMT Active Site Tryptic Peptides following Incubation of MGMT with *O*^6^-alkG-Containing ODNs

**Figure 1:**
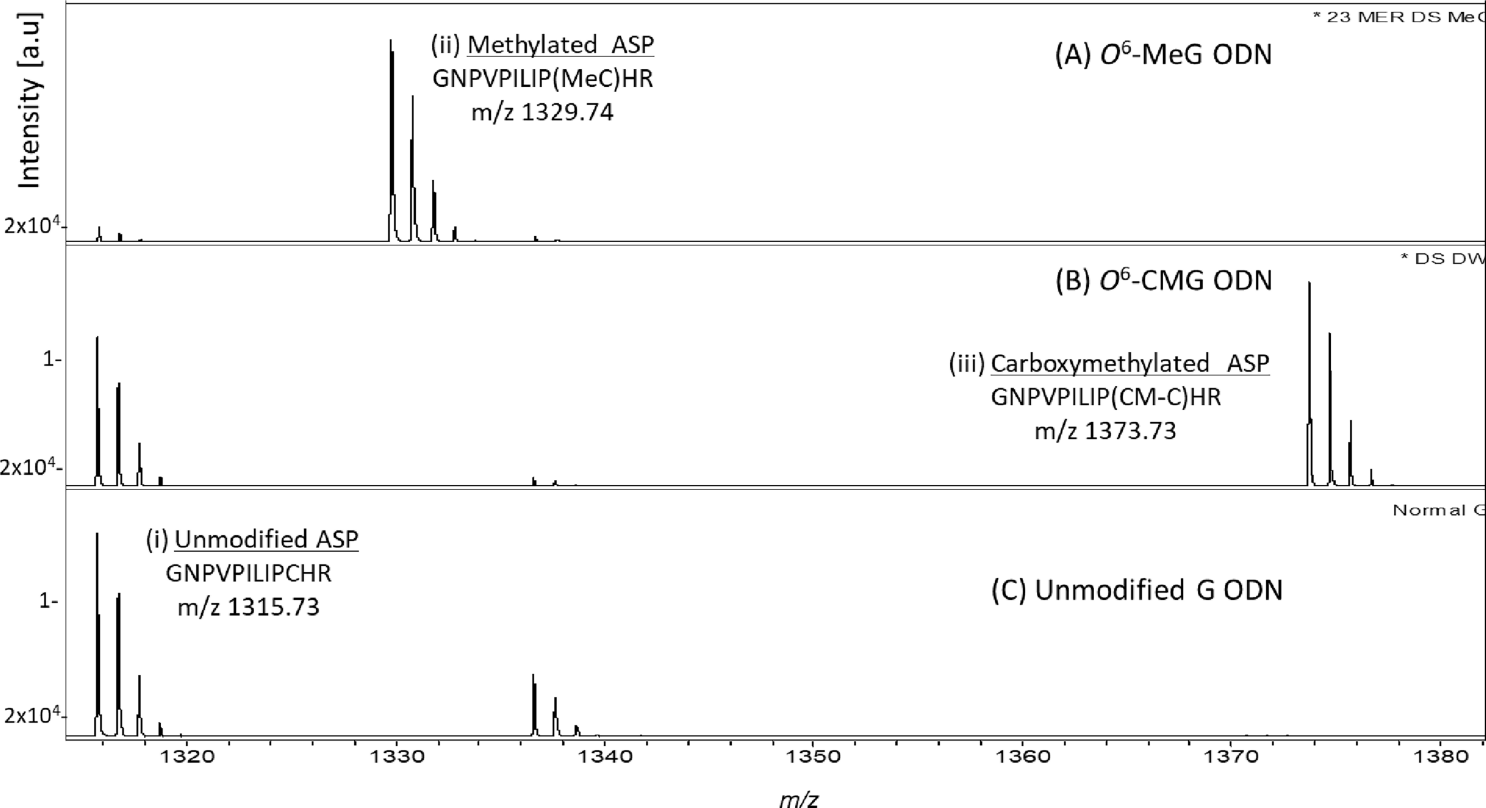
MALDI-ToF mass spectra of tryptic peptides of MBP-MGMT following its incubation with double-stranded 23-mer ODNs containing (A) *O*^6^-MeG, (B)*O*^6^-CMG, or (C) guanine. 2 pmol of active MBP-MGMT was incubated for 6 h at 37 °C with 20 pmol of 5′-GAA CTY CAG CTC CGT GCT GGC CC where Y is (A) *O*^6^-MeG, (B) *O*^6^-CMG, or (C) G and then digested with trypsin. Peak intensities are shown in arbitrary units on the *y*-axis, and the intensity scale is the same for all panels. The tryptic peptides detected included unmodified MGMT-ASP (GNPVPILIPCHR, 136–147) *m*/*z* 1315.73, methylated MGMT-ASP (GNPVPILIP(Me-C)HR, 136–147) *m*/*z* 329.74, and carboxymethylated MGMT-ASP (GNPVPILIP(CM-C)HR, 136–147) *m*/*z* 1373.73. The peak at *m*/*z* 1336.544 is an MBP peptide (SYEEELAKDPR, 332–342).

Qualitative MS analysis of tryptic fragments of MBP-MGMT following incubation with *O*^6^-MeG- and *O*^6^-CMG-containing DS ODNs confirmed the transfer of the methyl and carboxymethyl groups from *O*^6^-MeG and *O*^6^-CMG, respectively, to MGMT: modified MGMT-ASPs were detected by the ions formed from methylated MGMT-ASP (*m*/*z* 1329.7 [M + H]^+^) and carboxymethylated MGMT-ASP (*m*/*z* 1373.7 [M + H]^+^) (Figure 1A,B, respectively). In addition, MS analysis of tryptic digests of MBP-MGMT incubated with DS control G ODNs generated multiple MGMT and MBP peptides and, as expected, only the nonalkylated MGMT-ASP (*m*/*z* 1315.73 [M + H]^+^), as shown in Figure 1C. Ethyl, propyl, benzyl, pyridyloxobutyl, and hydroxyethyl groups were also transferred to the active site cysteine of MGMT from ODNs containing *O*^6^-EtG, *O*^6^-PrG, *O*^6^-BnG, *O*^6^-pobG, and *O*^6^-HOEtG, respectively (Figure s2 panel A). In contrast, incubation of MGMT with ODNs containing damage not known to be repaired by MGMT such as *N*^6^-hydroxypropyl-2,6-diaminopurine, *O*^6^-aminoethylG, *O*^6^-methyladamantylG, or 2,6-diaminopurine confirmed as expected the lack of transfer of the corresponding alkyl group to MGMT (Figure s2 panel B).

The presence of methylcysteine in the MGMT ASP was further confirmed after incubation of *O*^6^-MeG-containing SS ODNs with His-MGMT, with high confidence detection and identification of the methylated ASP by the fragments obtained with MS^E^. The ASP was identified as a high confidence peptide with a mass error <2.8 ppm and via tandem mass spectrometry (Table s1, Figures s3–s6).

We then investigated the detection limit of the MALDI-ToF MS assay by using serial dilutions of a synthesized methylated ASP. Linear correlations (amount vs peak area) with *R*^2^ values of 0.9985 and 0.9998 were obtained for unlabeled methylated and heavy isotope (^13^C^15^N proline)-labeled methylated MGMT-ASP standards, respectively (Figure s7). The lower limit of quantification of unlabeled (*m*/*z* = 1329.74) and ^13^C^15^N proline-labeled (*m*/*z* = 1335.74) methylated MGMT-ASP was found to be <20 fmol with a signal/noise ratio of >16.

### MALDI-ToF MS Analysis of MGMT Active Site Peptides following Incubation of MGMT with Methylated CT-DNA

Following His-MGMT incubation with methylated CT-DNA, MALDI-ToF MS analysis of tryptic peptides demonstrated the presence of methylated MGMT-ASP (GNPVPILIPMe-CHR, amino acid residues 136–147) in MGMT tryptic peptides. Figure s7A shows the region of the mass spectra of his-MGMT incubated with methylated CT-DNA (containing 0.125, 0.25, 0.5 pmol of *O*^6^-MeG/mg methylated CT-DNA) showing both unmodified and methylated MGMT-ASP ions at *m*/*z* 1315.72 and 1329.74, respectively. The observed PAs of methylated MGMT-ASP showed a linear correlation with levels of *O*^6^-MeG adducts (Figure s7B), and the LLOQ for MGMT-based detection of *O*^6^-MeG in methylated CT-DNA was <0.05 pmol *O*^6^-MeG per mg CT-DNA. The optimized approach was verified by considering the recovery of methylated MGMT-ASP following His-MGMT incubation with methylated CT-DNA using the ^13^C^15^N internal standard. Based on the ratio of PA-methylated ASP to PA Me-ASP STD recovery values of methylated MGMT-ASP fragments were 39.8 ± 0.9%, 41 ± 3.6%, and 48 ± 7.5% (mean ± SD; *n* = 3) following His-MGMT incubation with methylated CT-DNA that contained 100, 200, and 400 fmol of *O*^6^-MeG, respectively.

### Identification of *O*^6^-alkylG Adducts in Human Colorectal DNA

**Figure 2:**
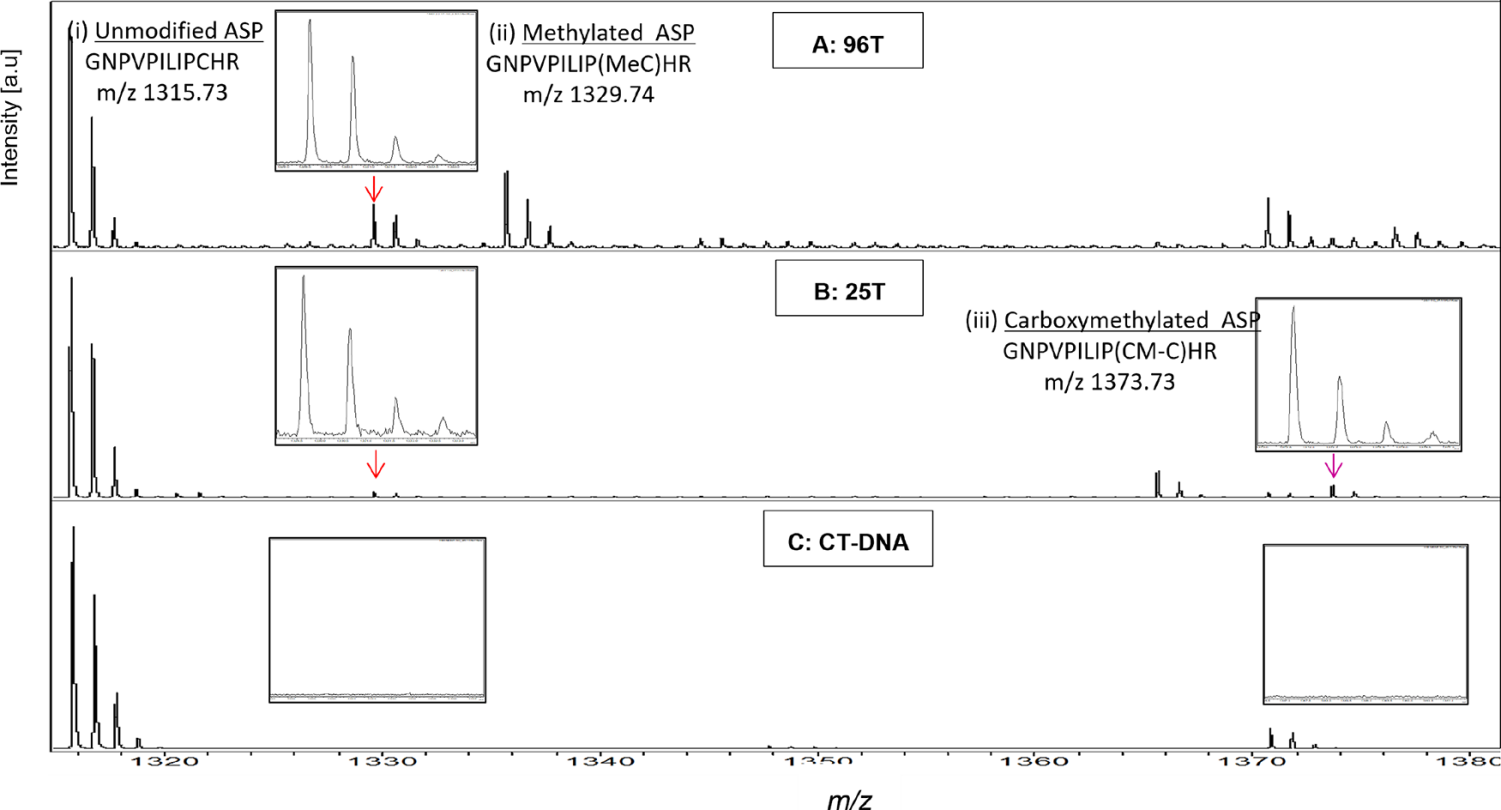
MALDI-ToF mass spectra analysis of alkylated ASPs present after incubating his-MGMT with human DNA. His-MGMT (50 pmol) was incubated with 2 mg of human colorectal DNA samples (A) 96T, (B) 25T, or (C) unmodified CT-DNA. Peak intensities are shown in arbitrary units on the *y*-axis, and the intensity scale is the same for all panels. S/*N* > 10 for all detected alkylated MGMT ASPs. The tryptic peptides (residues 136–147) identified were (i) unmodified MGMT-ASP (GNPVPILIPCHR; *m*/*z* 1315.73), (ii) methylated MGMT-ASP (GNPVPILIPMe-CHR; *m*/*z* 1329.74), and (iii) carboxymethylated MGMT-ASP (GNPVPILIPCM-CHR; *m*/*z* 1373.73).

Both methylated and carboxymethylated MGMT-ASPs were detected following the incubation of his-MGMT with colorectal DNA. Figure 2 shows the mass spectra for His-MGMT incubated with two different human colorectal tumor DNA samples (96T and 25T) or with unmodified CT-DNA. Both methylated and carboxymethylated MGMT ASPs (*m*/*z* 1329.74 [M + H]+ and *m*/*z* 1373.73 [M + H]+, respectively) were detected in these two samples, indicating the presence of the respective adducts in both DNA samples.

### Quantification of *O*^6^-alkylG Adducts in Human Colorectal DNA

Following identification of *O*^6^-alkG adducts present in human colorectal DNA samples, MGMT tryptic digests were spiked with ^13^C^15^N proline-labeled methylated MGMT-ASP and *O*^6^-MeG was quantified. *O*^6^-MeG was present in all human colorectal DNA samples analyzed at concentrations that ranged from 6.7 to 11.1 and from 5.1 to 78.2 nmol *O*^6^-MeG/mol dG for normal and tumor DNA, respectively (Table 1). For the two patients for which we had paired normal and tumor DNA, the levels of *O*^6^-MeG in the tumor DNA were higher. Table 1 also shows the concentrations of *O*^6^-MeG adducts in the same human colorectal DNA samples quantified previously using an HPLC-RIA:^12^ there was a significant correlation between the results of the two assays, *r* = 0.86 (*p* = 0.014).

**Table 1:** *O*^6^-MeG and *O*^6^-CMG Levels in CR DNA

**ID**	**CR tissue type**	**nanomol of***O*^**6**^**-alkyl adduct per mole of dG (mean ± SD** (*n* = 3))			*O*^6^**-CMG**/*O*^**6**^**-MeG**
		*O*^**6**^**-MeG**	*O*^**6**^**-MeG**	*O*^**6**^**-CMG**	
		**ASP/MALDI-ToF**	**HPLC/RIA**^a^^,^^b^	**ASP/MALDI-ToF^b^**	**ASP/MALDI-ToF**
19	normal	6.7 ± 0.3	NA	ND	
19	tumor	20.7 ± 1.8	NA	68.2 ± 5.7	3.3
25	tumor	19.2 ± 0.9	25	26.4 ± 2.8	1.4
31	tumor	20.2 ± 1.3	20	41.0 ± 2.2	2.0
39	tumor	5.1 ± 0.4	NA	5.2 ± 0.2	1.0
44	normal	11.1 ± 0.8	5	ND	
44	tumor	21.8 ± 1.4	5	ND	
50	tumor	47.5 ± 6.9	48	48.8 ± 3.1	1.0
74	tumor	21.4 ± 0.8	26	21.5 ± 2.3	1.0
79	tumor	19.6 ± 0.9	19	26.8 ± 2.2	1.4
96	tumor	78.2 ± 7.9	NA	34.5 ± 2.7	0.4

a Levels previously detected by HPLC-RIA.^16^

b NA = not available; ND = not detected.

The levels of *O*^6^-CMG in human colorectal tumor DNA ranged from 5.2 to 68.2 nmol of *O*^6^-CMG/mol of dG (Table 1). There was no association between *O*^6^-MeG and *O*^6^-CMG levels in human colorectal DNA (*P* = 0.93), and the *O*^6^-CMG/*O*^6^-MeG ratio ranged from 0.44 to 3.30.

### Evidence for a Human *O*^6^-alkG Adductome

**Figure 3:**
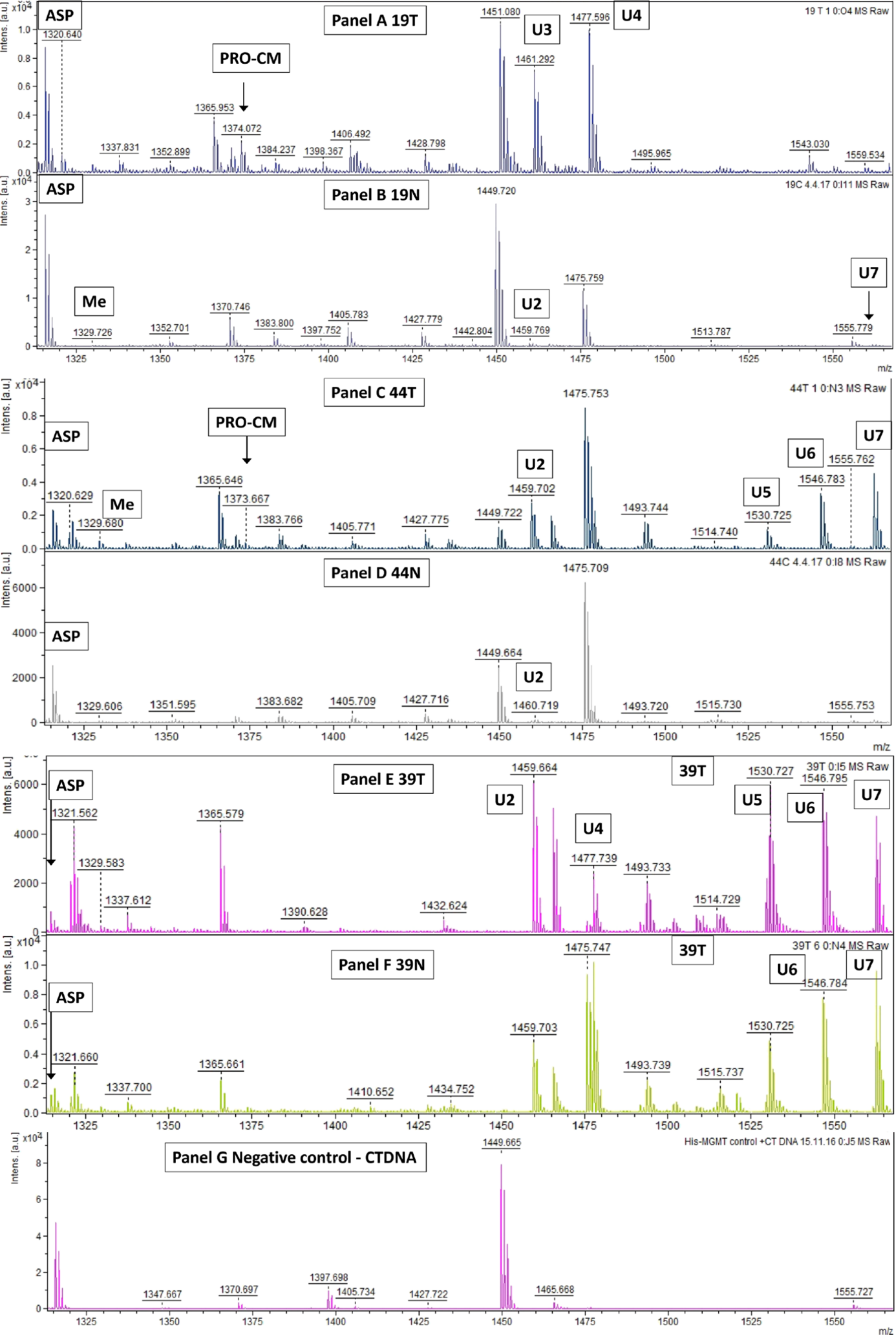
MALDI-TOF mass spectra analysis of alkylated ASPs present after incubating his-MGMT with paired normal and tumor colorectal DNA. His-MGMT (50 pmol) was incubated with 2 mg of human colorectal DNA samples isolated from paired tumor (T) or normal (N) tissues numbered 19, 44, and 39 and unmodified CT-DNA. Peak intensities are shown in arbitrary units on the *y*-axis, and the intensity scale is the same for all panels. S/*N* > 10 for all detected alkylated MGMT ASPs. These included unidentified (U) ASPs with *m*/*z* values of 1459.7 (labeled U2) found in four samples, 1461.7 (labeled U3) in one sample, 1477.7 (labeled U4) in two samples, 1530.7 (labeled U5) in two samples, 1546.7 (labeled U6) in three samples, and 1555.7 (labeled U7) in four samples.

In addition to methylated and carboxymethylated ASPs, a number of other ASPs were detected at varying frequencies following incubation of MGMT with paired colorectal normal and tumor DNA samples (Figure 3). These included ASPs with *m*/*z* values of 1459.7, 1461.7, 1477.7, 1530.7, 1546.7, and 1555.7, which correspond to alkyl group modifications of mass between 144 and 240. These modifications have not yet been identified, but, in one sample, a peptide with an *m*/*z* of 1359.7 was detected and we hypothesized that this ASP was the result of the transfer of the hydroxyethyl (HOEt) group from *O*^6^-hydroxyethyl guanine in the DNA. In support of this, when a synthetic DS ODN containing *O*^6^-HOEtG was incubated with MGMT, an ASP ion with the same *m*/*z* value was detected (data not shown).

## Discussion

In the present work, we have used MGMT to irreversibly transfer alkyl groups from *O*^6^-alkGs in DNA to the active site cysteine residue of the protein and, following tryptic digestion, detected the resulting alkylated ASPs by MALDI-ToF MS. This method was developed and validated using DS ODNs containing *O*^6^-MeG and *O*^6^-CMG and was able to detect ASPs at levels as low as 50 fmol. The potential scope of the method was demonstrated by using the ODNs containing other *O*^6^-alkGs having widely different alkyl group structures. Further validation came from the analysis of human colorectal DNA, which found levels of *O*^6^-MeG that were directly comparable to those found in the same human DNA samples by using HPLC-RIA.

Analysis of CR DNA by this approach revealed the presence of not only *O*^6^-MeG and *O*^6^-CMG in normal and tumor tissue but also a putative *O*^6^-HOEtG and a number of other adducts that are currently unidentified. Previous studies have shown that alkyl adducts are present in human DNA from both normal and tumor tissue. For example, *O*^6^-MeG has been detected in DNA from both normal and tumor tissue from the GI tract,^19^*O*^6^-CMG has been detected in colon tumor samples,^23^ and methyl DNA phosphate adducts have been detected in human lung tumor tissue and adjacent normal tissue.^24^ In this study, human DNA samples showed varied patterns in the *O*^6^-alkG presence, suggesting that individuals have very different patterns of exposure to the responsible AA and/or different levels of expression of endogenous MGMT perhaps as a result of MGMT methylation in tumors.^12,15^ The source of these AAs is currently unknown, but humans are likely exposed to a plethora of environmental and dietary alkylating agents as well as endogenously formed N-nitroso compounds.^25,26^ These are very likely to generate numerous mutagenic *O*^6^-alkG DNA adducts that may result in a complex array of genomic modifications, some or all of which may contribute to colorectal carcinogenesis. It is also clear that determining the levels of a single *O*^6^-alkG adduct will significantly underestimate human exposure and likely that with increased sensitivity, the method will detect many more unknown alkyl groups. Nevertheless, these data clearly demonstrate the presence of an alkyl adductome that is yet to be completely characterized. The importance of such an alkyl adductome is confirmed by the identification of an AA mutation signature in CR cancers.^5^

The method that we described has two advantages over other current approaches to measure alkyl DNA adducts. First, *O*^6^-alkGs are detected independent of the nature of the alkyl group enabling the detection of unknown *O*^6^-alkGs in contrast to, for example, antibody-based methods that can only detect known *O*^6^-alkGs.^42^ Furthermore, the potential cross-reactivity of *O*^6^-alkG-derived antibodies with different adducts is avoided. Analysis of human DNA in this study clearly indicates that multiple *O*^6^-alkG adducts can be detected in one sample at the same time. Second, by targeting *O*^6^-alkGs, this approach focuses on adducts that are biologically relevant because of their pro-mutagenicity and pro-carcinogenicity,^2,6,43^ in contrast to other adductomic approaches that detect a wide range of DNA adducts, including some that have little biological significance.^32,33^ The assay, however, depends absolutely on the ability of MGMT to repair *O*^6^-alkGs by alkyl transfer to ASP cysteine. The range of MGMT repairable *O*^6^-alkGs in DNA remains unknown although a range of different *O*^6^-alkGs in DNA are known substrates, though not necessarily repaired at the same rate.^44^ Optimization of the repair reaction (e.g., by increasing incubation time and the ratio of MGMT to DNA) may increase the ability of the assay to detect poorly repaired substrates, but interestingly, a modified ASP consistent with the presence of *O*^6^-HOEt in DNA was detected, and *O*^6^-HOEt is a poor MGMT substrate in ODNs.^37^ Further indications of the range of alkyl groups that are MGMT substrates comes from work with pseudosubstrates (*O*^6^-alkGs in the form of free guanine bases) whose alkyl group is removed by MGMT.^45^ More than 75 pseudosubstrates with alkyl group masses ranging from 40 to 319 Da have been synthesized and have been shown to inhibit MGMT activity potentially as a result of alkyl group transfer though competitive inhibition cannot be ruled out (Margison Pers. commun.) Where this has been assessed, the same pseudosubstrate following its incorporation into an ODN is significantly more potent at MGMT inactivation.^37,46^*O*^6^-alkGs in DNA that are not MGMT substrates, such as *O*^6^-methyladamantylG, were not detected by this assay. At present, neither the nature nor the biological significance of MGMT-irreparable *O*^6^-alkGs is known, but if they are not repaired by other DNA repair pathways such as nucleotide excision repair^47^ and are also mutagenic, they may also be deleterious to cells. However, associations between downregulation of MGMT via promoter methylation and increased GC-AT transition mutations in human DNA and an alkylating agent mutation signature^10−13^ clearly demonstrate that MGMT substrates are important in human mutagenesis and such adducts would be detected by our methodology.

This current study used MALDI-ToF MS analysis for detection of alkylated MGMT-ASPs, and the evidence of the detection of the alkylated peptides following MGMT incubation with human colorectal DNA was based on the following three criteria: (1) *m*/*z* of detected ions, (2) recorded change in the molecular weight of the ASP (observed mass shift), and (3) S/*N* > 10. This strategy allows accurate and reproducible quantification of *O*^6^-alkG adducts in terms of quantifying detected alkylated peptides using isotopically labeled (^13^C^15^N) internal standards at low cost. Furthermore, the assay offers the necessary sensitivity that is critical to detect the inherently low level of *O*^6^-alkG adducts. Though we do not have tandem MS data to confirm the identity of the putative alkylated peptides detected in MGMT digest following incubation with human DNA, levels of *O*^6^-MeG detected by this assay were correlated with those detected by an HPLC-RIA. Furthermore, the ready availability of DNA containing *O*^6^-alkG adducts using the methods that we have described previously^37^ allows in principle the generation of any alkylated ASP standard following incubation of the DNA with MGMT. In addition, our novel assay analyzed relatively large amounts of human colorectal DNA, which, although it facilitates the detection of low levels of *O*^6^-alkG, may not always be available. To overcome the limitation of low DNA amounts, we are currently investigating the use of targeted MS assays that rely on reaction monitoring, e.g., multiple reaction monitoring (MRM) on a tandem quadrupole instrument and/or parallel reaction monitoring (PRM) on the Thermo Orbitrap series.

In summary, our current work, coupling the action of MGMT with MALDI-ToF MS analysis, provides a novel, sensitive approach for the simultaneous detection of overall DNA *O*^6^-guanine alkylation damage in human DNA and, where standards are available, allows the level of known individual adducts to be determined. The sensitivity of the method is limited by the amounts of DNA that can be extracted and analyzed, the ability of MGMT to remove the alkyl groups from the *O*^6^-alkGs, and the potential for extremely rare adducts that are potential MGMT substrates that might be present at levels that are less than the lowest limit of quantitation. Furthermore, future development of the methodology should enable the identification of previously unknown *O*^6^-alkG adducts in human DNA and hence a comprehensive description of the *O*^6^-alkG adductome and its potential contribution to the etiology of human cancer.

## Supporting Information Available

ESI MS spectra of modified 23-mer ODNs; MALDI-ToF MS analysis of tryptic digests of MGMT incubated with different *O*^6^-alkyl-containing ODNs; LC-Vion IMS QToF analysis of His-MGMT tryptic digest following incubation with control SS ODN and SS ODN containing *O*^6^-MeG; quantitation of MGMT ASPs; characterization of ASPs formed following incubation with SS ODN containing *O*^6^-MeG (PDF)

## References

[ref1] Lijinsky W. Chemistry and biology of N-nitroso compounds; Cambridge Monographs on Cancer Research, Cambridge University Press: Cambridge, 1992.

[ref2] Shrivastav N.; Li D.; Essigmann J. M. Chemical biology of mutagenesis and DNA repair: cellular responses to DNA alkylation. Carcinogenesis 2010, 31, 59–70. 10.1093/carcin/bgp262. 19875697 PMC2802671

[ref3] International Agency for Research on Cancer. Review of Human Carcinogens; IARC Monograph: Lyon France, 2012. http://monographs.iarc.fr/ENG/Monographs/vol100E/mono100E-9.pdf.

[ref4] Alexandrov L. B.; Nik-Zainal S.; Wedge D. C.; Aparicio S. A.; Behjati S.; Biankin A. V.; et al. Signatures of mutational processes in human cancer. Nature 2013, 500, 415–421. 10.1038/nature12477. 23945592 PMC3776390

[ref5] Gurjao C.; Zhong R.; Haruki K.; Li Y. Y.; Spurr L. F.; Lee-Six H.; et al. Discovery and Features of an Alkylating Signature in Colorectal Cancer. Cancer Discovery 2021, 11, 2446–2455. 10.1158/2159-8290.CD-20-1656. 34140290 PMC8487940

[ref6] Kaina B.; Christmann M.; Naumann S.; Roos W. P. MGMT: key node in the battle against genotoxicity, carcinogenicity and apoptosis induced by alkylating agents. DNA Repair 2007, 6, 1079–1099. 10.1016/j.dnarep.2007.03.008. 17485253

[ref7] Margison G. P.; Santibáñez Koref M. F. *O*^6^-alkylguanine-DNA alkyltransferase: its role in carcinogenesis and chemotherapy. Bioessays 2002, 24, 255–266. 10.1002/bies.10063. 11891762

[ref8] Esteller M.; Hamilton S. R.; Burger P. C.; Baylin S. B.; Herman J. G. Inactivation of the DNA repair gene *O*^6^-methylguanine-DNA methyltransferase by promoter hypermethylation is a common event in primary human neoplasia. Cancer Res. 1999, 15, 793–797. 10029064

[ref9] Bouras E.; Karakioulaki M.; Bougioukas K. I.; Aivaliotis M.; Tzimagiorgis G.; Chourdakis M. Gene promoter methylation and cancer: An umbrella review. Gene 2019, 710, 333–340. 10.1016/j.gene.2019.06.023. 31202904

[ref10] Esteller M.; Risques R. A.; Toyota M.; Capella G.; Moreno V.; Peinado M. A.; et al. Promoter hypermethylation of the DNA repair gene *O*^6^-methylguanine-DNA methyltransferase is associated with the presence of G:C to A:T transition mutations in p53 in human colorectal tumorigenesis. Cancer Res. 2001, 61, 4689–4692. 11406538

[ref11] Wolf P.; Hu Y. C.; Doffek K.; Sidransky D.; Ahrendt S. A. *O*^6^-methylguanine-DNA methyltransferase promoter hypermethylation shifts the p53 mutational spectrum in non-small cell lung cancer. Cancer Res. 2001, 61, 8113–8117. 11719438

[ref12] Yin D.; Xie D.; Hofmann W. K.; Zhang W.; Asotra K.; Wong R.; et al. DNA repair gene *O*^6^-methylguanine-DNA methyltransferase promoter hypermethylation associated with decreased expression and G:C to A:T mutations of p53 in brain tumours. Mol. Carcinog. 2003, 36, 23–31. 10.1002/mc.10094. 12503076

[ref13] Knijnenburg T. A.; Wang L.; Zimmermann M. T.; Chambwe N.; Gao G. F.; Cherniack A. D.; et al. Genomic and Molecular Landscape of DNA Damage Repair Deficiency across the Cancer Genome Atlas. Cell Rep. 2018, 23, 239–254. 10.1016/j.celrep.2018.03.076. 29617664 PMC5961503

[ref14] Binabaj M. M.; Bahrami A.; ShahidSales S.; Joodi M.; Joudi Mashhad M.; Hassanian S. M.; et al. The prognostic value of MGMT promoter methylation in glioblastoma: A meta-analysis of clinical trials. J. Cell Physiol. 2018, 233, 378–386. 10.1002/jcp.25896. 28266716

[ref15] Christmann M.; Kaina B. Epigenetic regulation of DNA repair genes and implications for tumor therapy. *Mutat. Res./Rev*. Mutat. Res. 2019, 780, 15–29. 10.1016/j.mrrev.2017.10.001. 31395346

[ref16] De Bont R.; van Larebeke N. Endogenous DNA damage in humans: a review of quantitative data. Mutagenesis 2004, 19, 169–185. 10.1093/mutage/geh025. 15123782

[ref17] Li Y.; Hecht S. S. Metabolic Activation and DNA Interactions of Carcinogenic N-Nitrosamines to Which Humans Are Commonly Exposed. Int. J. Mol. Sci. 2022, 23, 4559 10.3390/ijms23094559. 35562949 PMC9105260

[ref18] Risk assessment of *N*-nitrosamines in food. EFSA J. 2023, 21, e07884 10.2903/j.efsa.2023.7884. 36999063 PMC10043641

[ref19] Hall C. N.; Badawi A. F.; O’Connor P. J.; Saffhill R. The detection of alkylation damage in the DNA of human gastrointestinal tissues. Br. J. Cancer 1991, 64, 59–63. 10.1038/bjc.1991.239. 1854628 PMC1977320

[ref20] Wilson V. L.; Weston A.; Manchester D. K.; Trivers G. E.; Roberts D. W.; Kadlubar F. F.; et al. Alkyl and aryl carcinogen adducts detected in human peripheral lung. Carcinogenesis 1989, 10, 2149–2153. 10.1093/carcin/10.11.2149. 2805234

[ref21] Palli D.; Saieva C.; Coppi C.; Del Giudice G.; Magagnotti C.; Nesi G.; et al. *O*^6^-alkylguanines, dietary N-nitroso compounds, and their precursors in gastric cancer. Nutr. Cancer 2001, 39, 42–49. 10.1207/S15327914nc391_6. 11588901

[ref22] Lewin M. H.; Bailey N.; Bandaletova T.; Bowman R.; Cross A. J.; Pollock J.; et al. Red meat enhances the colonic formation of the DNA adduct *O*^6^-carboxymethyl guanine: implications for colorectal cancer risk. Cancer Res. 2006, 66, 1859–1965. 10.1158/0008-5472.CAN-05-2237. 16452248

[ref23] Hemeryck L. Y.; Decloedt A. I.; Vanden Bussche J.; Geboes K. P.; Vanhaecke L. High resolution mass spectrometry-based profiling of diet-related deoxyribonucleic acid adducts. Anal. Chim. Acta 2015, 892, 123–131. 10.1016/j.aca.2015.08.019. 26388482

[ref24] Ma B.; Villalta P. W.; Hochalter J. B.; Stepanov I.; Hecht S. S. Methyl DNA phosphate adduct formation in lung tumor tissue and adjacent normal tissue of lung cancer patients. Carcinogenesis 2019, 40, 1387–1394. 10.1093/carcin/bgz053. 30873516 PMC6875899

[ref25] Gushgari A. J.; Halden R. U. Critical review of major sources of human exposure to N-nitrosamines. Chemosphere 2018, 210, 1124–1136. 10.1016/j.chemosphere.2018.07.098. 30208538

[ref26] Bartsch H.; Ohshima H.; Pignatelli B.; Calmels S. Endogenously formed *N*-nitroso compounds and nitrosating agents in human cancer etiology. Pharmacogenetics 1992, 2, 272–277. 10.1097/00008571-199212000-00005. 1339085

[ref27] Tricker A. R.; Pfundstein B.; Kälble T.; Preussmann R. Secondary amine precursors to nitrosamines in human saliva, gastric juice, blood, urine and faeces. Carcinogenesis 1992, 13, 563–568. 10.1093/carcin/13.4.563. 1576707

[ref28] Fan P.; Li L.; Rezaei A.; Eslamfam S.; Che D.; Ma X. Metabolites of Dietary Protein and Peptides by Intestinal Microbes and their Impacts on Gut. Curr. Protein Pept. Sci. 2015, 16, 646–654. 10.2174/1389203716666150630133657. 26122784

[ref29] Klapacz J.; Pottenger L. H.; Engelward B. P.; Heinen C. D.; Johnson G. E.; Clewell R. A.; et al. Contributions of DNA repair and damage response pathways to the non-linear genotoxic responses of alkylating agents. Mutat. Res. Rev. Mutat. Res. 2016, 767, 77–91. 10.1016/j.mrrev.2015.11.001. 27036068 PMC4818947

[ref30] Povey A. C.; Cooper D. P. The development,validation and application of a ^32^P-postlabelling assay to quantify *O*^6^-methylguanine in human DNA. Carcinogenesis 1995, 16, 1665–1669. 10.1093/carcin/16.7.1665. 7614705

[ref31] Kraus A.; McKeague M.; Seiwert N.; Nagel G.; Geisen S. M.; Ziegler N.; et al. Immunological and mass spectrometry-based approaches to determine thresholds of the mutagenic DNA adduct *O*^6^-methylguanine in vivo. Arch. Toxicol. 2019, 93, 559–572. 10.1007/s00204-018-2355-0. 30446773

[ref32] Balbo S.; Hecht S. S.; Upadhyaya P.; Villalta P. W. Application of a high-resolution mass-spectrometry-based DNA adductomics approach for identification of DNA adducts in complex mixtures. Anal. Chem. 2014, 86, 1744–1752. 10.1021/ac403565m. 24410521 PMC3982966

[ref33] Totsuka Y.; Watanabe M.; Lin Y. New horizons of DNA adductome for exploring environmental causes of cancer. Cancer Sci. 2021, 112, 7–15. 10.1111/cas.14666. 32978845 PMC7780056

[ref34] Watson A. J.; Middleton M. R.; McGown G.; Thorncroft M.; Ranson M.; Hersey P.; et al. *O*^6^-methyl- guanine-DNA methyltransferase depletion and DNA damage in patients with melanoma treated with Temozolomide alone or with lomeguatrib. Br. J. Cancer 2009, 100, 1250–1256. 10.1038/sj.bjc.6605015. 19367283 PMC2676560

[ref35] Pearson S.; Wharton S.; Watson A.; Begum G.; Butt A.; Glynn N.; et al. A novel DNA damage recognition protein in S.pombe. Nucleic Acids Res. 2006, 34, 2347–2354. 10.1093/nar/gkl270. 16679453 PMC1458281

[ref36] Watson A. J.; Margison G. P. *O*^6^-Alkylguanine-DNA Alkyltransferase Assay. Methods Mol. Biol. 2000, 152, 49–61. 10.1385/1-59259-068-3:49. 10957968

[ref37] Shibata T.; Glynn N.; McMurry T. B.; McElhinney R. S.; Margison G. P.; Williams D. M. Novel synthesis of *O*^6^-alkylguanine containing oligodeoxyribonucleotides as substrates for the human DNA repair protein, *O*^6^-methylguanine DNA methyltransferase (MGMT). Nucleic Acids Res. 2006, 34, 1884–1891. 10.1093/nar/gkl117. 16609128 PMC1435717

[ref38] Räz M. H.; Dexter H. R.; Millington C. L.; van Loon B.; Williams D. M.; Sturla S. J. Bypass of Mutagenic *O*^6^-Carboxymethylguanine DNA Adducts by Human Y-and B-Family Polymerases. Chem. Res. Toxicol. 2016, 29, 1493–1503. 10.1021/acs.chemrestox.6b00168. 27404553

[ref39] Wilkinson O. J.; Latypov V.; Tubbs J. L.; Millington C. L.; Morita R.; Blackburn H.; et al. Alkyltransferase-like protein (Atl1) distinguishes alkylated guanines for DNA repair using cation-π interactions. Proc. Natl. Acad. Sci. U. S. A. 2012, 109, 18755–18760. 10.1073/pnas.1209451109. 23112169 PMC3503161

[ref40] Senthong P.; Millington C. L.; Wilkinson O. J.; Marriott A. S.; Watson A. J.; Reamtong O.; et al. The nitrosated bile acid DNA lesion *O*^6^- carboxymethylguanine is a substrate for the human DNA repair protein *O*^6^-methylguanine-DNA methyltransferase. Nucleic Acids Res. 2013, 41, 3047–3055. 10.1093/nar/gks1476. 23335782 PMC3597670

[ref41] An overview of the principles of MSE the engine that drives MS performance; https://www.waters.com/webassets/cms/library/docs/720004036en.pdf. Accessed 22/10/2023.

[ref42] Santella R. M. Immunological Methods for Detection of Carcinogen-DNA damage in humans. Cancer Epidemiol. Biomarkers Prev. 1999, 8, 733–739. 10498391

[ref43] Margison G. P.; Santibáñez Koref M. F.; Povey A. C. Mechanisms of carcinogenicity/chemotherapy by *O*^6^-methylguanine. Mutagenesis 2002, 17, 483–487. 10.1093/mutage/17.6.483. 12435845

[ref44] Coulter R.; Blandino M.; Tomlinson J. M.; Pauly G. T.; Krajewska M.; Moschel R. C.; et al. Differences in the rate of repair of *O*^6^-alkylguanines in different sequence contexts by *O*^6^-alkylguanine DNA alkyltransferase. Chem. Res. Toxicol. 2007, 20, 1966–1971. 10.1021/tx700271j. 17975884

[ref45] McElhinney R. S.; McMurry T. B. H.; Margison G. P. *O*^6^-alkylguanine-DNA alkyltransferase inactivation in cancer chemotherapy. Mini Rev. Med. Chem. 2003, 3, 471–485. 10.2174/1389557033487980. 12769698

[ref46] Luu K. X.; Kanugula S.; Pegg A. E.; Pauly G. T.; Moschel R. C. Repair of oligodeoxyribonucleotides by *O*^6^- alkylguanine-DNA alkyltransferase. Biochemistry 2002, 41, 8689–8697. 10.1021/bi025857i. 12093287

[ref47] Taira K.; Kaneto S.; Nakano K.; Watanabe S.; Takahashi E.; Arimoto S.; et al. Distinct pathways for repairing mutagenic lesions induced by methylating and ethylating agents. Mutagenesis 2013, 28, 341–350. 10.1093/mutage/get010. 23446177 PMC3630523

