# Supplementary material for: Mass Spectrometric Analysis of the Active Site Tryptic Peptide of Recombinant O6-Methylguanine-DNA Methyltransferase Following Incubation with Human Colorectal DNA Reveals the Presence of an O6-Alkylguanine Adductome
Source: Chem Res Toxicol. 2023 Nov 20;36(12):1921–9. doi: 10.1021/acs.chemrestox.3c00207 (PMC10731659; doi:10.1021/acs.chemrestox.3c00207)
Supplement: Supplementary file 1 — tx3c00207_si_001.pdf [file tx3c00207_si_001.pdf]

Supplementary Information

Mass spectrometric analysis of the active site tryptic peptide of recombinant *O*^{6}-methylguanine-DNA methyltransferase following incubation with human colorectal DNA reveals the presence of an *O*^{6}-alkylguanine adductome.

Rasha Abdelhady[1,ǂ], Pattama Senthong[1,◊], Claire E. Eyers[2,Δ], Onrapak Reamtong[2,Π], Elizabeth Cowley [1], Luca Cannizzaro[2], Joanna Stimpson[2], Kathleen Cain[2], Oliver J. Wilkinson[3,⌂], Nicholas H. Williams [3], Perdita E. Barran[2], Geoffrey P. Margison[1], David M. Williams[3], Andrew C. Povey[1*]

[1] Epidemiology and Public Health Group, Division of Population Health, Health Services Research and Primary Care, School of Health Sciences, Faculty of Biology, Medicine and Health, University of Manchester, Manchester, M13 9PL, UK.
[2] Department of Chemistry and Manchester Institute of Biotechnology, University of Manchester, Manchester, M1 7DN, UK.
[3] Centre for Chemical Biology, Department of Chemistry, Sheffield Institute for Nucleic Acids, University of Sheffield, S3 7HF, UK.

Currently at
[ǂ] Faculty of Pharmacy, Fayoum University, Fayoum, 63514, Egypt.
[◊] Faculty of Science and Industrial Technology, Prince of Songkla University, Surat Thani Campus, Muang, Surat Thani, Thailand 84000.
[Δ] Centre for Proteome Research, Institute of Systems, Molecular & Integrative Biology, University of Liverpool, Liverpool, L69 7ZB, UK
[Π] Department of Molecular Tropical Medicine and Genetics, Faculty of Tropical Medicine, Mahidol University, Bangkok, Thailand 10400.
[⌂] Elizabeth Blackwell Institute, School of Biochemistry, University of Bristol BS8 1UH, UK.

Corresponding author:
Dr Andrew Povey, Ellen Wilkinson Building, University of Manchester, UK. M13 9PL.
Phone: 44 161 275 5232; e-mail; apovey@manchester.ac.uk

Table of contents

Figure/Table	Title	Page
Figure s1	ESI MS spectra of modified 23-mer ODNs	S3
Figure s2	MALDI-ToF mass spectral analysis of tryptic digests of MGMT incubated with different O^6-alkylG containing ODNs.	S4
Figure s3	LC-Vion IMS QToF analysis of purified His-MGMT after in-solution tryptic digestion.	S5
Figure s4	LC-Vion IMS QToF analysis of His-MGMT tryptic digest following incubation with SS ODN containing O^6-MeG.	S6
Figure s5	LC-Vion IMS QToF analysis of His-MGMT tryptic digest following incubation with control SS ODN (not containing O^6-MeG).	S7
Figure s6	LC-Vion IMS QTof analysis of His-MGMT tryptic digest following incubation with control SS ODN spiked with synthetic methylated ASP.	S8
Figure s7	Quantitation of MGMT ASPs.	S9
Table s1	ODNs(G) +His-MGMT shows the identification of the carbamidomethylated form of ASP with a mass error of 3 ppm and 12 matched first generation primary ions	S10
Table s2	ODNs(O^6-MeG)+His-MGMT shows the identification of the methyl form of ASP with a mass error of 3 ppm and 15 matched first generation primary ions, the identification of the carbamido-methylated form of ASP has a mass error of 3 ppm and 8 matched first generation primary ions.	S11
Table s3	ODNs(G)+His-MGMT+Spiked MeASP indicate the methyl form of the spiked ASP with a mass error of 2.4 ppm and 17 matched first generation primary ions while the identification of the carb-amidomethylated form of ASP has a mass error of 2.7 ppm and 7 matched first generation primary ions.	S12

Panel A

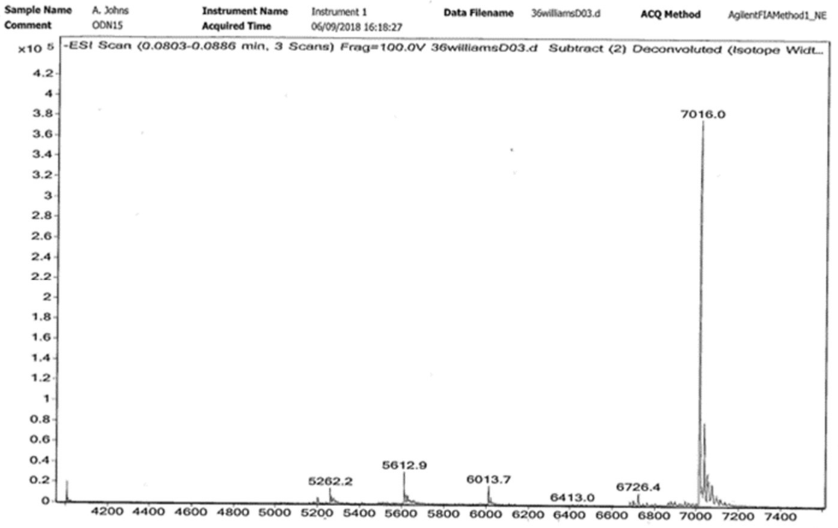

Panel B

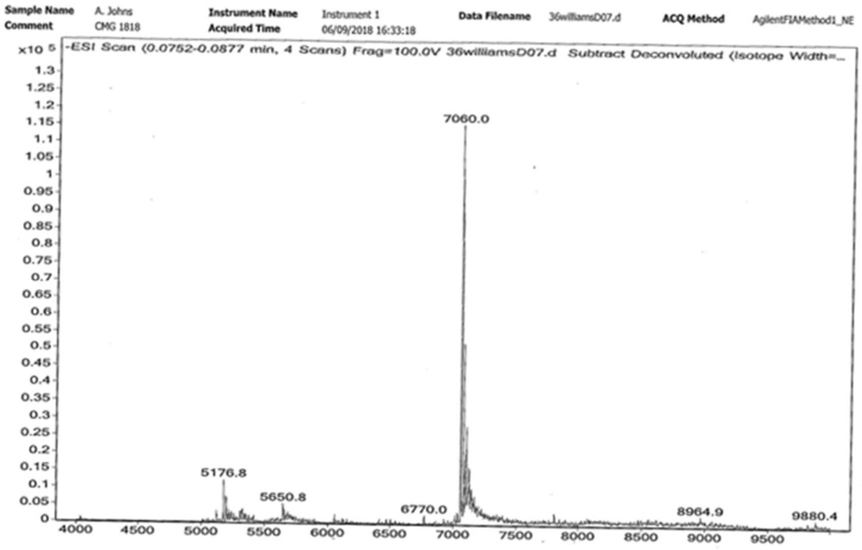

Figure s1 ESI MS spectra of modified 23-mer ODNs
Spectra were obtained on an Agilent Technologies 6530 accurate mass LC-MS QToF
ESI-MS of 5'-GAA CTY CAG CTC CGT GCT GGC CC [Y= *O*^{6}-MeG (panel A), Y= *O*^{6}-CMG (panel B).
Calculated masses (respectively) = 7015.5, 7059.5

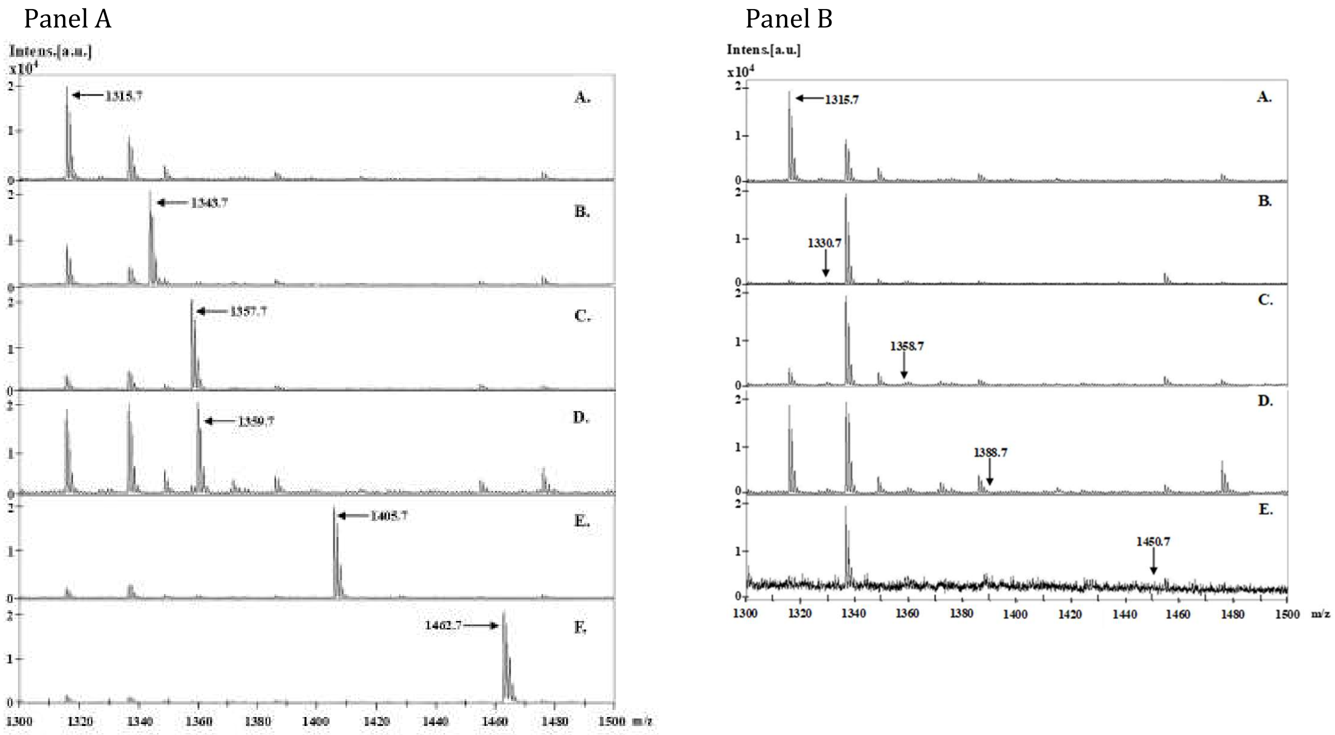

Figure s2 MALDI-ToF mass spectral analysis of tryptic digests of MGMT incubated with different *O*⁶-alkylG containing ODNs.
Single-stranded ODNs (5'-SIMA-GCCATGXCTAGTA) where X represents various O^6-alkylGs, were incubated with MGMT proteins for 1 h at 37 °C. Negative controls contained no ODNs. Trypsin (1 µg; ratio of MGMT: trypsin, 50:1) was added and the reaction was incubated overnight at 37 °C. The digestion was terminated with the addition of 0.1% formic acid. MALDI-ToF analyses were performed on a Bruker UltraflexTM (Bruker Daltonics, Bremen, Germany).

A: MALDI-ToF mass spectral analysis of tryptic digests of MGMT indicating the locations and masses of the fragments expected if alkyl group transfer occurred with ODNs containing: **A**. G, **B**. O^6-EtG, **C**. O^6-PrG, **D**. O^6-HOEtG, **E**. O^6-BenzylG, and **F**. O^6-PyridyloxobutylG.

B: MALDI-ToF mass spectral analysis of tryptic digests of MGMT indicating the locations and masses of the fragments expected if alkyl group transfer occurred with ODNs containing: **A**. Guanine, **B**. 2,6-diaminopurine, **C**. O^6-aminoethylG, **D**. N^6-hydroxypropyl-2,6-diaminopurine and **E**. O^6-methyladamantylG. In none of these cases was there any convincing evidence of alkyl or other group transfer.

A) HPLC elution profile of His-MGMT after tryptic digestion

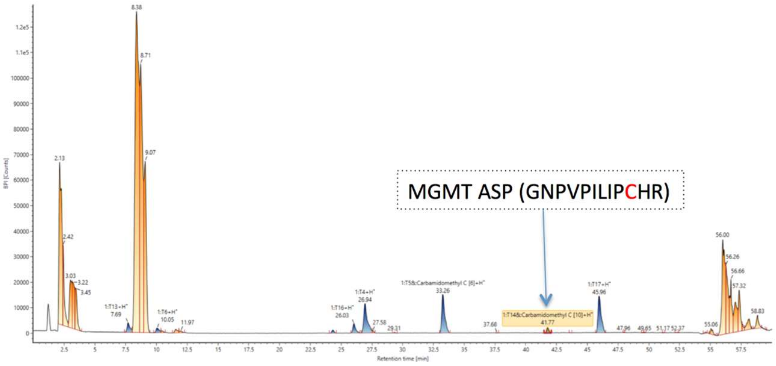

B) Fragmentation pattern of MGMT Me-ASP

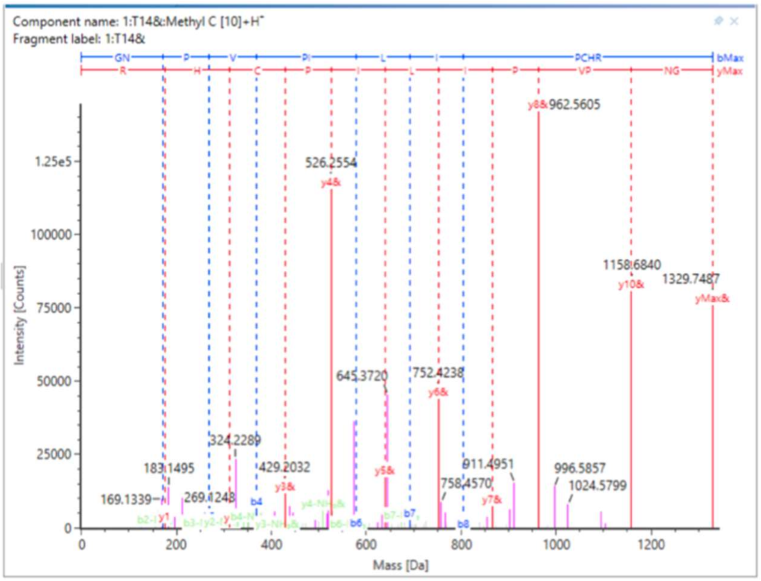

Figure s3 LC-Vion IMS QToF analysis of purified His-MGMT after in-solution tryptic digestion.

A. HPLC elution profile of His-MGMT after tryptic digestion.

BPI (base peak ion) chromatogram showing the elution profile with peaks of the digested peptides, including the one specific for MGMT-ASP at 41.77 min. Blue peaks are identified peptides while orange peaks are unidentified. Please see materials and methods section for HPLC details. The carbamidomethyl C modification is due to alkylation by iodacetamide during the in-solution digestion procedure.

B. Fragmentation pattern of MGMT ASP.

Identification of MGMT-ASP with its fragmentation pattern, showing the intensities, Y axis, and the Mass (Da), X axis, of all the fragments obtained by the Vion IMS QToF from the ASP at 41.77 min, whose sequence is in Panel A.

A) HPLC elution profile (zoom) of trypsin digested His-MGMT after incubation with SS ODNs containing O^6-MeG

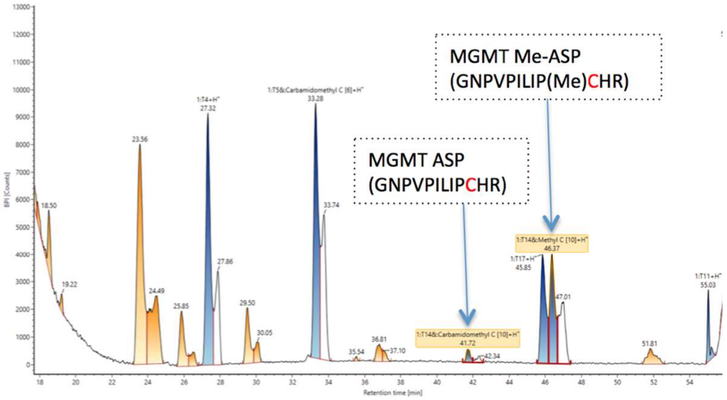

B) Fragmentation pattern of MGMT Me-ASP

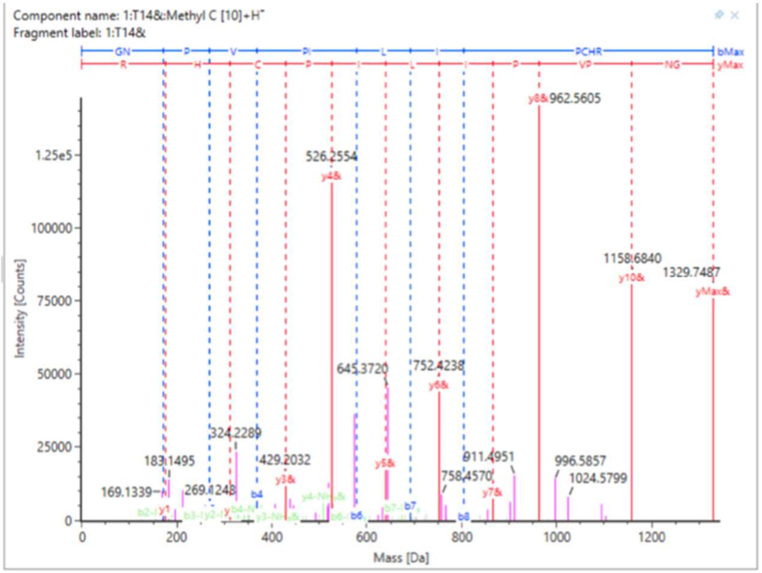

C) Fragmentation pattern of unreacted MGMT ASP after carbamidomethylation

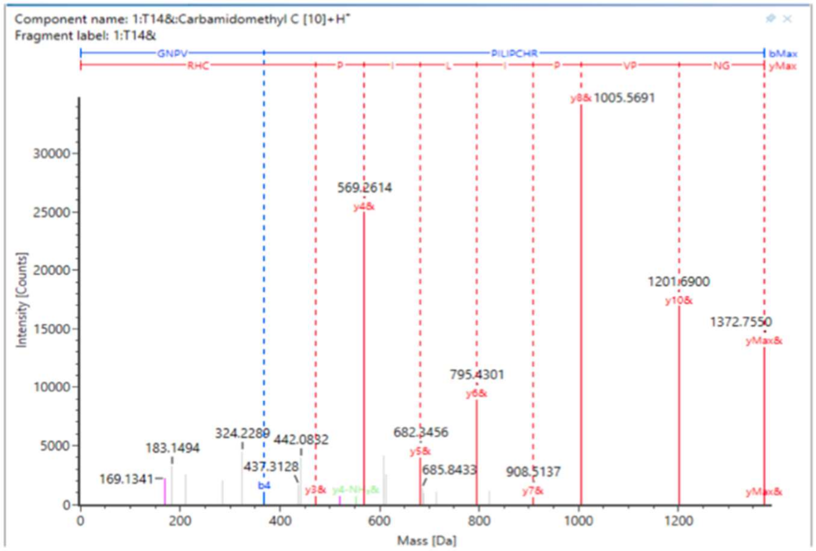

Figure s4 LC-Vion IMS QToF analysis of His-MGMT tryptic digest following incubation of 50 pmol His-MGMT with 37.5 nmol SS ODN containing O^6-MethylG. **A: HPLC elution profile (zoom) of trypsin digested His-MGMT after incubation with SS ODNs containing O^6-MeG:** BPI chromatogram showing the elution profile between 14-58 min with peaks of the digested peptides, indicating a specific peak of the methylated MGMT-ASP (retention time 46.37 min) indicating transfer of the methyl group from the O^6-MeG containing SS ODN to MGMT. The specific peak of the carbamidomethylated MGMT-ASP (retention time 41.72 min), reveals a small fraction of His-MGMT that did not react with the O^6-MeG containing SS ODN. Blue peaks are identified peptides while orange peaks are unidentified. See materials and methods section for HPLC details. **B: Fragmentation pattern of MGMT Me-ASP:** Identification of MGMT-ASPs with fragmentation patterns after incubation with ODNs (O^6-MeG) showing the intensities, Y axis, and the Mass (Da), X axis, of all the fragments obtained by the Vion IMS QToF from the methylated fractions of MGMT-ASP. **C: Fragmentation pattern of unreacted MGMT ASP after carbamidomethylation.** Identification of MGMT-ASP with fragmentation patterns after carbamidomethylation, due to alkylation of unmodified his-MGMT by iodoacetamide of the unreacted fraction of MGMT-ASP.

A) HPLC elution profile (zoom) of trypsin digested His-MGMT after incubation with SS ODNs not containing O^6-MeG

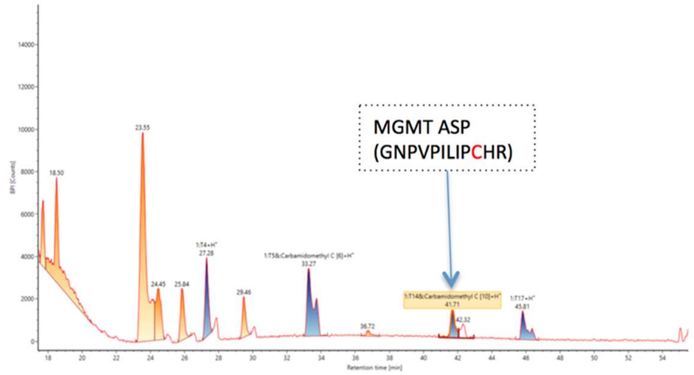

Figure s5 LC-Vion IMS QToF analysis of His-MGMT tryptic digest following incubation with control SS ODN (not containing *O*^{6}-MeG).

SS ODN not containing *O*^{6}-MeG (37.5 nmol) as negative control was incubated with His-MGMT (50 pmol).

A: HPLC elution profile (zoom) of trypsin digested His-MGMT after incubation with SS ODNs not containing *O*^{6}-MeG: BPI chromatogram showing the elution profile between 14-58 min with peaks of the digested peptides, indicating a specific peak of the carbamidomethylated MGMT-ASP (retention time 41.71 min), indicating, as expected, the absence of methylation as His-MGMT was incubated with control SS ODN lacking *O*^{6}-MeG. Consequently, the ASP identified from the tryptic digestion of His-MGMT was all found as carbamidomethyl C. Blue peaks are identified peptides while orange peaks are unidentified. See materials and methods section for HPLC details.

A) HPLC elution profile (zoom) of trypsin digested His-MGMT after incubation with SS ODNs not containing O^{6}-MeG spiked with synthesized methylated ASP

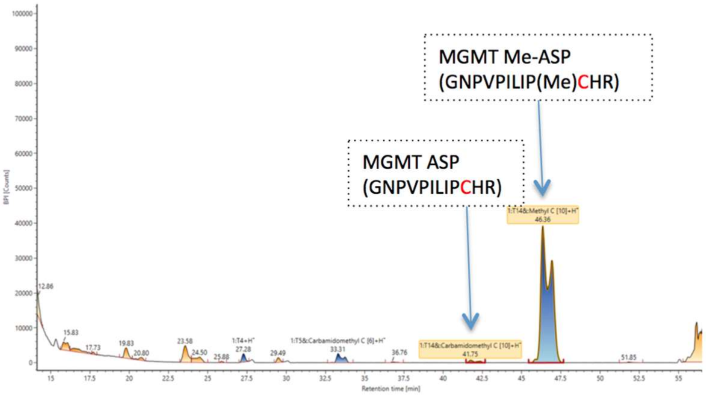

B) Fragmentation pattern of MGMT spiked with synthetic Me-ASP

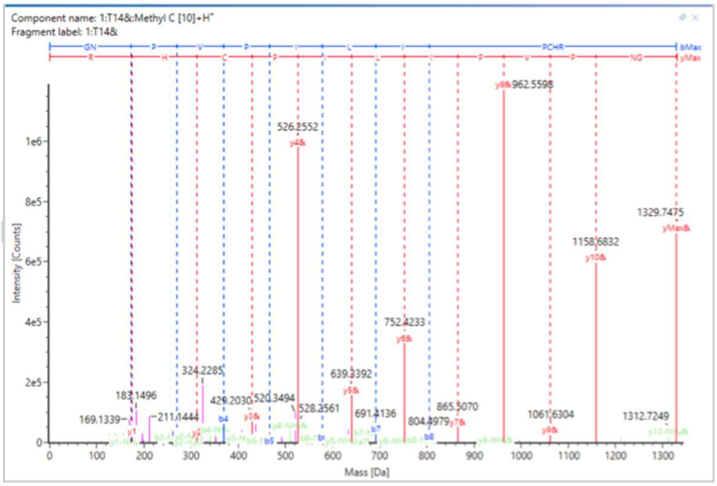

Figure s6 LC-Vion IMS QTof analysis of His-MGMT tryptic digest following incubation with control SS ODN spiked with synthetic methylated ASP.

Control SS ODN (37.5 nmol) was incubated with His-MGMT (50 pmol). Methylated synthetic ASP (112 nmol) was spiked in as a positive control.

A: HPLC elution profile (zoom) of trypsin digested His-MGMT after incubation with SS ODNs not containing *O*^{6}-MeG, spiked with synthesised methylated ASP. BPI chromatogram showing the elution profile between 14-58 min with peaks of the digested peptides, including specific peaks of both the spiked methylated synthetic MGMT-ASP (retention time 46.36 min), and of carbamidomethylated His-MGMT-ASP (retention time 41.75 min). Blue peaks are identified peptides while orange peaks are unidentified. See materials and methods section for HPLC details.

B: Fragmentation pattern of MGMT spiked with synthetic Me-ASP. Identification of the spiked synthetic Me-ASP with its fragmentation pattern showing the intensities, Y axis, and the Mass (Da), X axis, of all the fragments obtained by the Vion IMS QTof.

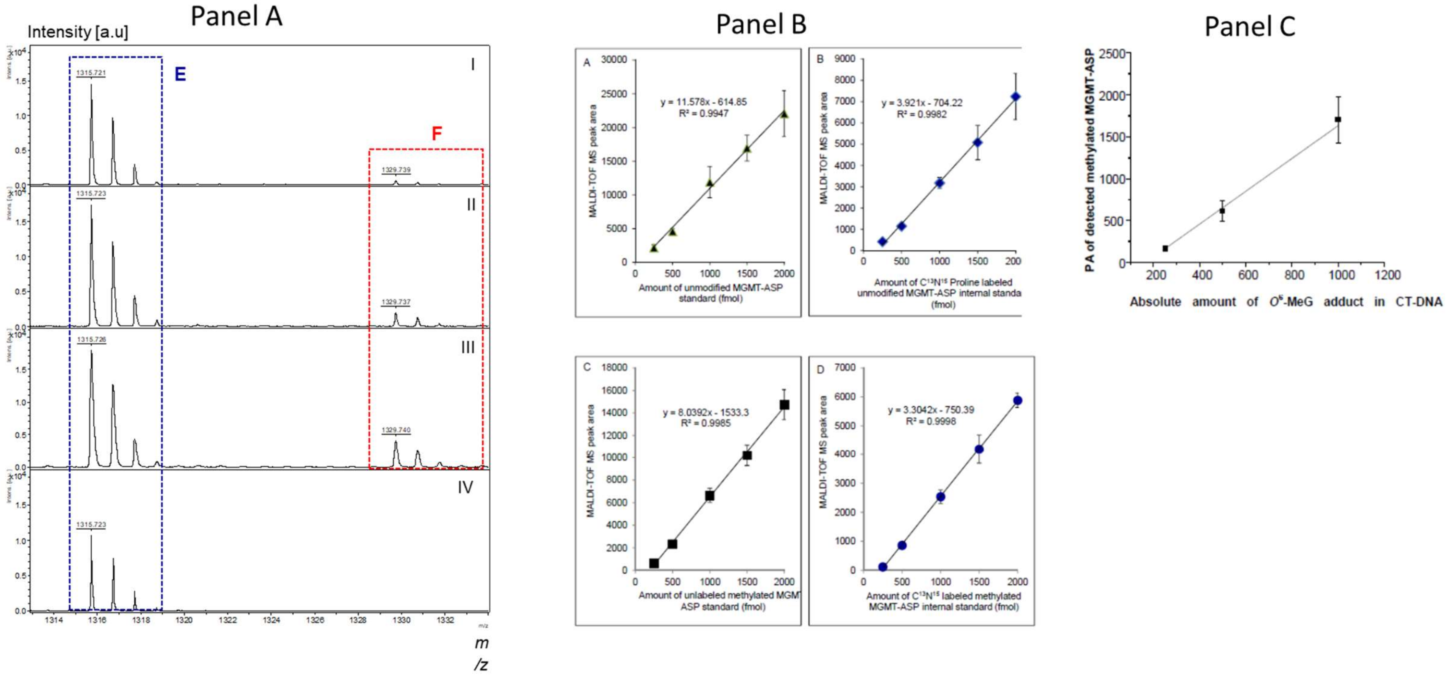

Figure s7 Quantitation of MGMT ASPs

A: MALDI-ToF MS analysis of ASPs: His-MGMT (50 pmol) was reacted with Temozolomide-modified CT-DNA (2 mg) that contained (I) 0.125, (II) 0.25 and (III) 0.5 pmol *O*^{6}-MeG per mg CT-DNA or with (IV) unmodified CT-DNA. His-MGMT was recovered using Ni-coated magnetic beads and treated *in situ* with trypsin. Peak intensities are shown in arbitrary units on the y-axis and max peak intensity is the same for all panels. No peak corresponding to methylated MGMT-ASP was detected in control unmodified CT-DNA; **B: Dose response curve of synthetic unmodified and methylated ASP as analysed by MALDI-ToF MS:** A Unlabelled unmodified MGMT-ASP standard; B $^{13}C^{15}N$ proline labelled unmodified MGMT-ASP internal standard; C Unlabelled methylated MGMT-ASP standard; D $^{13}C^{15}N$ proline labelled methylated MGMT-ASP internal standard; **C: Dose-response of methylated MGMT-ASP signal vs amount of *O*^{6}-MeG in methylated CT-DNA:** His-MGMT (50 pmol) was reacted with Temozolomide-modified CT-DNA (2 mg) that contained 0.25, 0.5 or 1 pmol *O*^{6}-MeG per mg CT-DNA. His-MGMT was recovered using Ni-coated magnetic beads and treated *in situ* with trypsin. Results are expressed as PA of methylated MGMT-ASP vs amount of adduct incubated with His-MGMT. No peaks corresponding to methylated MGMT-ASP were detected in control unmodified CT-DNA with S/N>10.

Table s1 ODNs(G) +His-MGMT shows the identification of the carbamidomethylated form of ASP with a mass error of 3 ppm and 12 matched first generation primary ions.

Protein name	Component name	Peptide	Modifiers	Sequence start	Sequence end	Observed m/z	Mass error (ppm)	Observed RT (min)	Response	Charge	Matched 1st Gen Primary Ions	% Matched 1st Gen Primary Ions (%)	Matched Primary Ions Flag	Assigned intensity (%)	% Peptide (%)	Primary Ions Intensity Flag	Alternate assignments
His-MGMT	1:T6+H^+	LLGK		55	58	215.656	3.74	17.85	181451.9	2	2	33.3	Best Match	88.0	100.0	Best Match	No
His-MGMT	1:T13+H^+	AVGGAMR		151	157	331.177	2.22	16.99	60061.7	2	1	8.3	Below Threshold	22.1	58.2	Best Match	No
His-MGMT	1:T8+H^+	QVLWK		119	123	337.206	2.69	25.87	105779.9	2	2	25.0	Best Match	12.3	100.0	Best Match	No
His-MGMT	1:T4+H^+	TTLDSPLGK		32	40	466.259	1.21	27.30	1135229.5	2	9	56.3	Best Match	91.8	69.5	Best Match	No
His-MGMT	1:T18+H^+	GAGATSGSPPAGR		216	228	543.271	0.71	8.24	65622.4	2	4	16.7	Best Match	38.1	62.8	Best Match	No
His-MGMT	1:T3-4+H^+	RTTLDSPLGK		31	40	544.310	2.26	25.23	69344.9	2	4	22.2	Best Match	24.1	100.0	Best Match	No
His-MGMT	1:T16+H^+	EWLLAHEGHR		188	197	416.549	2.76	29.35	140836.9	3	1	5.6	Below Threshold	9.8	100.0	Best Match	No
His-MGMT	1:T14&:Carbamidomethyl C [10]+H^+	GNPVPILIPCHR	Carbamidomethyl C [10]	158	169	458.257	3.07	41.71	821975.1	3	12	54.5	Best Match	82.4	59.0	Best Match	No
His-MGMT	1:T5&:Carbamidomethyl C [6]+H^+	LELSGCEQGLHEIK	Carbamidomethyl C [6]	41	54	538.272	1.87	33.29	1738072.3	3	15	57.7	Best Match	90.5	62.8	Best Match	No
His-MGMT	1:T17+H^+	LGKPGLGGSSGLAGAWLK		198	215	556.987	2.08	45.85	902079.5	3	20	58.8	Best Match	93.9	63.4	Best Match	No
His-MGMT	1:T15&:Carbamidomethyl C [3]+H^+	VVCSSGAVGNYSGGLAVK	Carbamidomethyl C [3]	170	187	862.935	0.07	33.38	581072.0	2	21	61.8	Best Match	92.3	66.4	Best Match	No
His-MGMT	1:T11+H^+	FGEVISYQQLAALAGNPK		130	147	636.009	2.27	55.04	289483.4	3	14	41.2	Best Match	81.0	100.0	Best Match	No

Table s2 ODNs(O^6-MeG)+His-MGMT shows the identification of the methyl form of ASP with a mass error of 3 ppm and 15 matched first generation primary ions, the identification of the carbamidomethylated form of ASP has a mass error of 3 ppm and 8 matched first generation primary ions.

Protein name	Component name	Peptide	Modifiers	Sequence start	Sequence end	Observed m/z	Mass error (ppm)	Observed RT (min)	Response	Charge	Matched 1st Gen Primary Ions	% Matched 1st Gen Primary Ions (%)	Matched Primary Ions Flag	Assigned intensity (%)	% Peptide (%)	Primary Ions Intensity Flag	Alternate assignments
His-MGMT	1:T6+H^+	LLGK		55	58	215.6556233	3.66	18.11	281077.1	2	3	50.0	Best Match	95.8	100.0	Best Match	No
His-MGMT	1:T13+H^+	AVGGAMR		151	157	661.3456169	0.92	8.09	113854.6	1	1	8.3	Below Threshold	14.0	100.0	Best Match	No
His-MGMT	1:T8+H^+	QVLWK		119	123	337.2058649	1.90	25.92	224200.6	2	3	37.5	Best Match	25.3	100.0	Best Match	No
His-MGMT	1:T4+H^+	TTLDSPLGK		32	40	466.2587414	0.77	27.32	2292062.5	2	10	62.5	Best Match	91.3	69.1	Best Match	No
His-MGMT	1:T18+H^+	GAGATSGSPPAGR		216	228	543.271205	1.59	8.19	97399.8	2	5	20.8	Best Match	78.7	58.1	Best Match	No
His-MGMT	1:T3-4+H^+	RTTLDSPLGK		31	40	544.3091703	0.43	25.27	143575.7	2	7	38.9	Best Match	33.3	100.0	Best Match	No
His-MGMT	1:T16+H^+	EWLLAHEGHR		188	197	416.5484384	2.23	29.27	170649.3	3	5	27.8	Best Match	61.3	100.0	Best Match	No
His-MGMT	1:T14&:Methyl C [10]+H^+	GNPVPILIPCHR	Methyl C [10]	158	169	443.9215407	3.07	46.38	1778777.6	3	15	68.2	Best Match	62.5	47.2	Best Match	No
His-MGMT	1:T14&:Carbamidomethyl C [10]+H^+	GNPVPILIPCHR	Carbamidomethyl C [10]	158	169	458.2568183	2.99	41.74	362542.2	3	8	36.4	Best Match	77.6	9.6	Best Match	No
His-MGMT	1:T5&:Carbamidomethyl C [6]+H^+	LELSGCEQGLHEIK	Carbamidomethyl C [6]	41	54	538.2720757	1.08	33.30	3948462.3	3	20	76.9	Best Match	84.1	62.3	Best Match	No
His-MGMT	1:T17+H^+	LGKPGLGGSSGLAGAWLK		198	215	556.986832	1.66	45.88	2105905.5	3	23	67.6	Best Match	81.7	60.6	Best Match	No
His-MGMT	1:T15&:Carbamidomethyl C [3]+H^+	VVCSSGAVGNYSGGLAVK	Carbamidomethyl C [3]	170	187	862.9347866	-0.75	33.39	1028840.1	2	26	76.5	Best Match	91.1	64.8	Best Match	No
His-MGMT	1:T11+H^+	FGEVISYQQLAALAGNPK		130	147	636.0080325	1.39	55.03	818687.0	3	20	58.8	Best Match	73.1	100.0	Best Match	No

Table s3 ODNs(G)+His-MGMT+Spiked MeASP indicate the methyl form of the spiked ASP with a mass error of 2.4 ppm and 17 matched first generation primary ions while the identification of the carbamidomethylated form of ASP has a mass error of 2.7 ppm and 7 matched first generation primary ions.

Protein name	Component name	Peptide	Modifiers	Sequence start	Sequence end	Observed m/z	Mass error (ppm)	Observed RT (min)	Response	Charge	Matched 1st Gen Primary Ions	% Matched 1st Gen Primary Ions (%)	Matched Primary Ions Flag	Assigned intensity (%)	% Peptide (%)	Primary Ions Intensity Flag	Alternate assignments
His-MGMT	1:T6+H$^+$	LLGK		55	58	215.6550023	0.77	17.69	61219.7	2	2	33.3333	Best Match	12.0	100.0	Best Match	No
His-MGMT	1:T8+H$^+$	QVLWK		119	123	337.2057949	1.69	25.84	63522.3	2	1	12.5	Below Threshold	11.6	100.0	Best Match	No
His-MGMT	1:T4+H$^+$	TTLDSPLGK		32	40	466.2588721	1.05	27.30	775787.5	2	8	50	Best Match	93.5	68.8	Best Match	No
His-MGMT	1:T18+H$^+$	GAGATSGSPPAGR		216	228	543.2709752	1.17	8.25	48013.9	2	4	16.6667	Best Match	70.9	60.2	Best Match	No
His-MGMT	1:T3-4+H$^+$	RTTLDSPLGK		31	40	544.3090868	0.28	25.00	38198.4	2	1	5.55556	Below Threshold	21.5	100.0	Best Match	No
His-MGMT	1:T14&:Methyl C [10]+H$^+$	GNPVPILIPCHR	Methyl C [10]	158	169	443.9212677	2.45	46.37	13935087.0	3	17	77.2727	Best Match	79.2	52.5	Best Match	No
His-MGMT	1:T14&:Carbamidomethyl C [10]+H$^+$	GNPVPILIPCHR	Carbamidomethyl C [10]	158	169	458.2567081	2.75	41.77	428702.5	3	7	31.8182	Best Match	83.4	1.6	Best Match	No
His-MGMT	1:T5&:Carbamidomethyl C [6]+H$^+$	LELSGCEQGLHEIK	Carbamidomethyl C [6]	41	54	538.2722711	1.45	33.33	1353125.0	3	15	57.6923	Best Match	89.3	62.4	Best Match	No
His-MGMT	1:T17+H$^+$	LGKPGLGGSSGLAGAWLK		198	215	556.986858	1.71	45.90	691180.8	3	13	38.2353	Best Match	88.0	59.3	Best Match	No
His-MGMT	1:T15&:Carbamidomethyl C [3]+H$^+$	VVCSSGAVGNYSGGLAVK	Carbamidomethyl C [3]	170	187	862.9353283	-0.13	33.42	353082.2	2	16	47.0588	Best Match	90.7	62.2	Best Match	No
His-MGMT	1:T11+H$^+$	FGEVISYQQLAALAGNPK		130	147	636.0088201	2.63	55.04	285447.1	3	15	44.1176	Best Match	57.5	100.0	Best Match	No
His-MGMT	1:T7&:Carbamidomethyl C [26]+H$^+$	GTSAADAVEVPAPAAVLGGPEPLMQCTAWLNAYFHQPEAIEEFPVPALHHPVFQQESFTR	Carbamidomethyl C [26]	59	118	1306.042326	1.92	55.21	52434.9	5	11	9.32203	Best Match	13.0	100.0	Best Match	No
